# The Progress of Surgery in 1890

**Published:** 1891-04

**Authors:** 


### The Progress of Surgery in 1890.

In the last year there has possibly been a slight lull in the accumulation of surgical discoveries, and yet we are unable to look back upon the progress of the year that has just expired without feeling that there is ample ground for congratulations to the medical profession upon the strides that have been made.

The great event of the year was the Medical Congress at Berlin, where the large attendance of men most prominent in our art and science from all parts of the civilized world brought together a wealth of facts and views that are now crystalizing into a valuable mass of information.

There has been noticed in the year gone by that reaction which was bound to follow the enthusiastic prevalence of intra-abdominal operations during the last few years. The matchless skill of the leaders in these procedures has taught us a technique that can scarcely be improved upon; while the number of operations performed has become less, through more perfect discrimination in the selection of cases absolutely requiring such measures. In the matter of operations for the relief of disorders due to inflammatory conditions of the vermiform appendix, the main points developed have been chiefly in the line of improved diagnosis, and in a general belief in the advisability of early interference.

The study of intestinal anastomosis, first evolved by Dr. Senn, has been taken up by a host of men, though, strange to say, its practical applications are still limited too much in our own country. The monograph written by Professor Senn upon the subject of the Diagnosis and Operative Treatment of Gunshot Wounds of the Stomach and Intestines, bids fair to become one of the classics of surgery for years to come. No better illustration of the progress made in this domain of surgery can be given than the brilliant results obtained by Professor Bernays in a series of seven cases of gunshot wounds of the abdomen, which formerly would have been regarded as hopeless, and left to die without surgical interference.

The methods of skin grafting and transplantation devised by Thiersch have proven their value in many hands during the past year, and now are fully admitted among the established surgical procedures of common occurrence.

Mr. Horsley's experiments in the transplantation of the thyroid gland of animals to the human subject have been followed by a practical application of the procedure, and bid fair to add one more to the many splendid results attained by this indefatigable experimenter.

In the surgery of the joints a more conservative tendency is becoming manifest; and in Germany especially, the injection of iodoform emulsions in tuberculous joints has been employed with much success as a substitute for more radical procedures.

Among the improvements brought about in the technique of special operative procedures, the bloodless method of amputation at the hip joint, devised by professor Wyeth, of New York, and performed with success by himself and others, takes high rank.

Operations upon the nerve-centres and the nerves themselves have greatly multiplied, and operations of un-heard-of magnitude have this year been reported, by which the brain and cord have become a far wider operative field than had ever been previously thought possible.

The discovery with which Koch startled the medical world a few months since has not been sufficiently developed to permit of positive deductions regarding its value in the various forms of surgical tuberculosis, although from present indications it will supersede all other methods for the treatment of lupus.

Such are a few of the results of the past year, and in this marvellous age of progress we are left to wonder at what we may have to chronicle for another year, though we are conscious that the last one has marked a decided era of improvement in our methods and knowledge.—*International Journal of Surgery.*

---

Cold in the Head. For cold in the head, while in the acute congestive stage, there is no better remedy than gelseminum. One good large dose, say 10 minims of the fluid extract, taken upon going to bed, will effectually dispose of this troublesome and uncomfortable affection. One dose is usually sufficient. *Medical Compend.*

Alveolar Abscess. An abscess is a circumscribed cavity containing pus, circumscribed by the formation of a soft membrane forming a sack, restraining the pus from passing into the surrounding tissue, causing it to seek the surface at the point of the least resistance; here parting the tissues, forming an escape called a festulous opening.

An alveolor abscess is, as the name indicates, an abscess originating within the alveolar walls. They are usually formed at the apex of the roots, the death of the pulp of one root only is sufficient to cause an abscess. Alveolar abscesses may arise from other causes than that of the decomposition of a tooth pulp. Any foreign matter, such as filling material, being forced through the apex of the root, calcic deposits within the walls of the alveolus, necrosed root or bone, impacted teeth, etc., may cause sufficient inflammation of the periosteum to produce an abscess, all the successive stages of inflammation being involved in the formation of an abscess, from irritation to suppuration.

Treatment required is first to gain free access to the diseased parts, and remove the cause. This can be done by opening up the canals, and if need be, by drilling through the gum and alveolus over the affected parts (in opening up the canals it is better to sacrifice good tooth structure than to attempt to work around corners and through too small a canal, being careful not to go through the side of the root) and remove all broken down and dead tissue. Then such remedies should be used as will incite a healthy action in the parts. For this purpose there is nothing better in our materia medica than peroxide of hydrogen and bichloride of mercury. Warren, Pathology.

Adulteration Laws in Russia.—The Russian Government has recently enacted some very stringent laws against the adulteration of food and drink. Any person guilty of adulterating any article of food will be liable to a fine of $200, or imprisonment for three months for the first offense, double this penalty for the second, and deprivation of all rights as a citizen for the third. What a sweeping revolution would follow the enactment of such a law in the United States. As a question of profit and loss, it would be simply stupendous.

